# Respiratory virus surveillance in the post-pandemic era: challenges and opportunities for dashboard-based public health action

**DOI:** 10.1186/s12919-026-00368-2

**Published:** 2026-03-16

**Authors:** Adrienne Halley, Caroline Schneeberger, Foekje F. Stelma, Aby Ba Diallo, Ombeline Jollivet, Bronke Boudewijns, Marie-Noëlle Billard, Julika Frome, Jean-Sebastien Casalegno, Katharina B. Lauer, Cédric Mahé, Erica Dueger, Marco Del Riccio, Alexandre Descamps, Anna Maisa, Siddhivinayak Hirve, Saverio Caini, Marta C. Nunes

**Affiliations:** 1 Netherlands Institute for Health Services Research (Nivel), Utrecht, the Netherlands; 2 Amsterdam University Medical Center (AUMC), Amsterdam, the Netherlands; 3 Center of Excellence in Respiratory Pathogens (CERP), Hospices Civils de Lyon (HCL) and Centre International de Recherche en Infectiologie (CIRI), Équipe Santé Publique, Épidémiologie Et Écologie Évolutive Des Maladies Infectieuses (PHE3ID), Inserm, ENS de Lyon, Université Claude Bernard Lyon, Lyon, France; 4 Sanofi Vaccine, Lyon, France; 5 Department of Pediatric Infectious Diseases and Immunology, Wilhelmina Children’s Hospital, University Medical Center Utrecht, Utrecht, Netherlands; 6 ReSViNET Foundation, Zeist, The Netherlands; 7 Laboratoire de Virologie, Centre National de Référence Des Virus Des Infections Respiratoires, Institut Des Agents Infectieux, Hospices Civils de Lyon, Lyon, France; 8 Airfinity Ltd, London, UK; 9 University of Duisburg-Essen, Essen, Germany; 10 Department of Health Sciences, University of Florence, Florence, Italy; 11 Directorate General for Health, Ministry of Health, Paris, France; 12 Department of Infectious Diseases, Santé Publique France, Saint-Maurice, France; 13 Global Influenza Programme, World Health Organization, Geneva, Switzerland

**Keywords:** Respiratory pathogen surveillance, Surveillance dashboards, Data integration, Data standardization, Data governance, Collaboration

## Abstract

Respiratory pathogen surveillance dashboards surged during the COVID-19 pandemic and have remained widely used tools for real-time data visualization in public health. While these dashboards offer timely, actionable insights for monitoring trends and decision-making, their rapid expansion has also highlighted persistent challenges related to governance, data accessibility, standardization, and sustainability. To explore these issues in depth, the Center of Excellence for Respiratory Pathogens (CERP) hosted a two-day workshop in Lyon, France. Experts representing a range of respiratory pathogen surveillance initiatives convened to share experiences, highlight successes, and discuss ongoing challenges. Key themes included the need for improved data quality, transparency, and standardization; sustainable IT infrastructure and staffing; greater access to underlying data; and alignment between dashboard objectives and user needs. Participants emphasized that broader governance and collaboration challenges strongly impact dashboard performance and interoperability. This report summarizes the valuable insights and subsequent actionable recommendations that emerged from the workshop, offering guidance to both developers and users of respiratory pathogen (or disease burden) dashboards. It aims to support the development of a more integrated, effective, and sustainable global respiratory surveillance ecosystem.

## Introduction

**Fig. 1:**
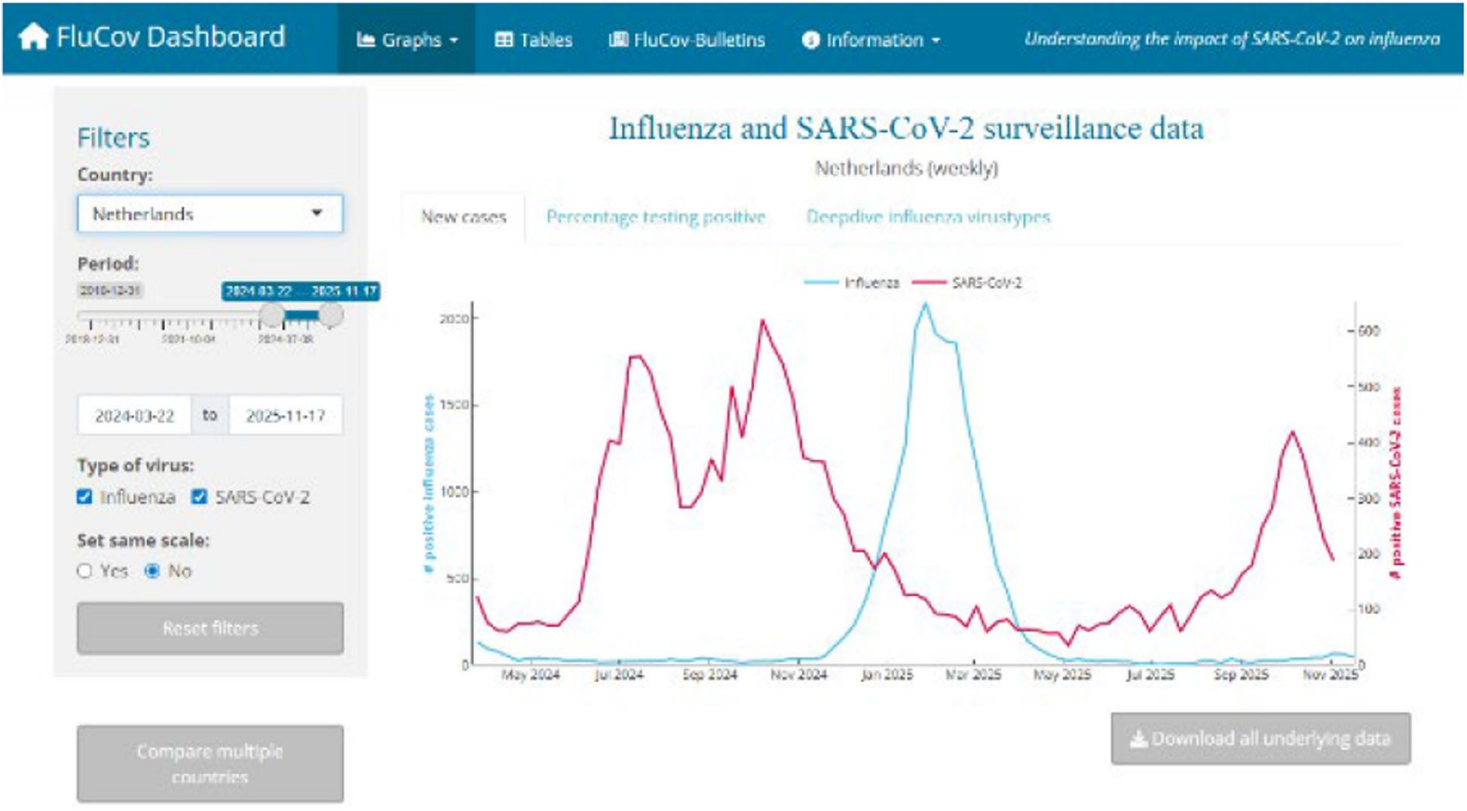
FluCov Dashboard. FluCov interactive dashboard available from Nivel website visualizing co-circulation of Influenza and SARS-CoV-2 (FluCov Dashboard | Nivel) (Inactive as of December 2025)

**Fig. 2:**
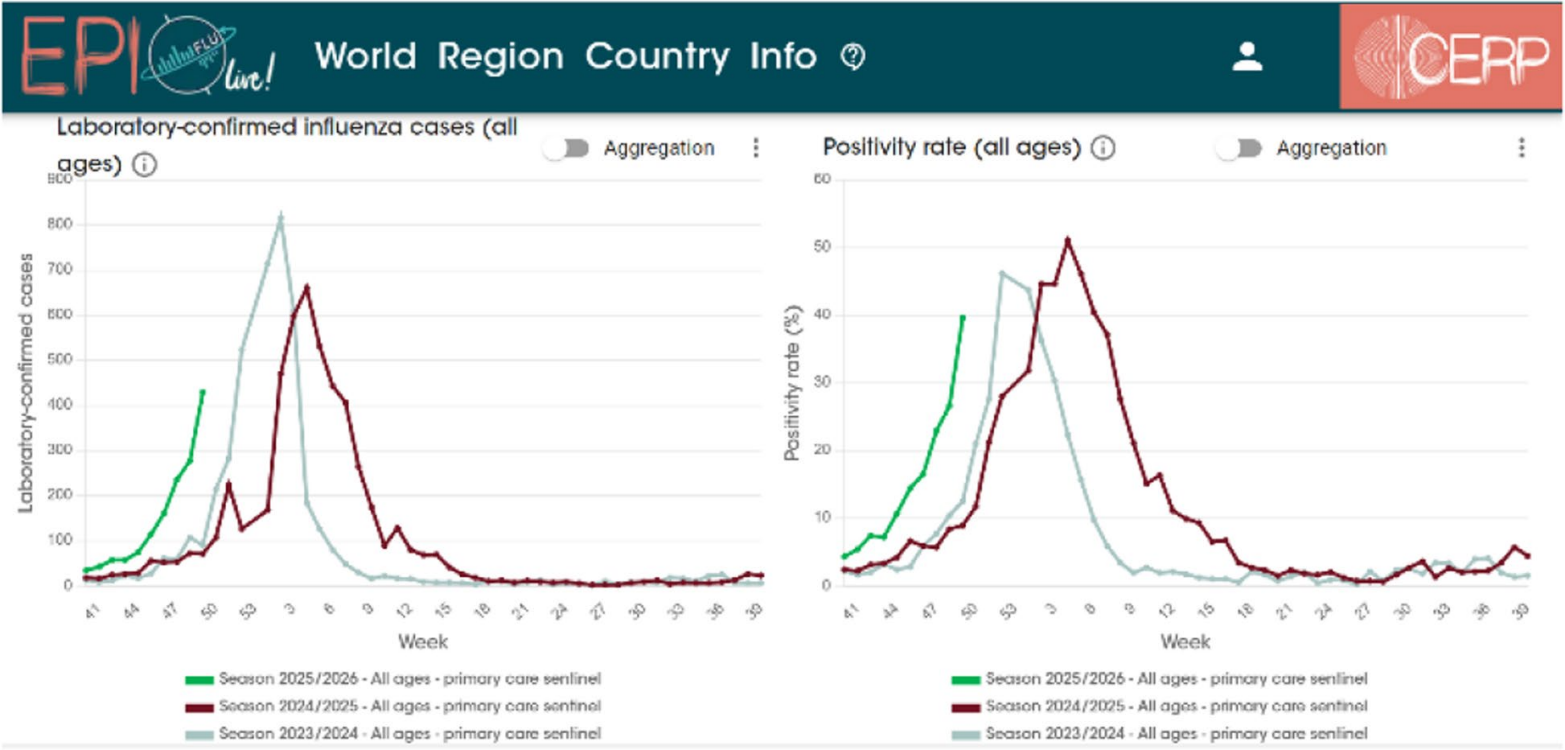
EPI Live! website and dashboard. Example of national influenza weekly-positivity rate across different seasons (CERP—EPI Live! Flu)

Respiratory pathogen surveillance requires the continuous, systematic collection, analysis, and interpretation of health and pathogen-related data to inform monitoring and public health decision-making [1]. The apparition of new viruses triggering or increasing risk of pandemic, e.g. COVID-19-, the persistent threat posed by (re)emerging influenza viruses with human pandemic potential (IVPPs), and the need to monitor epidemiological characteristics and trends during interpandemic periods to adapt or promote public health strategies, have highlighted the needs, strengths, and shortcomings of current surveillance systems [2]. During the COVID-19 pandemic, there was a rapid evolution in digital surveillance strategies and data visualization methods (see examples in Figs. 1 and 2). Aggregated data platforms and (near) real-time dashboards gained prominence, facilitating dissemination of comprehensive, complex information during crisis, and thereby, improved monitoring of disease trends, outbreak detection, vaccine development, research, and public health decision-making [3]. Although a temporary surge, the desire for and usefulness of these tools has persisted [4]. However, this rapid growth raised issues such as unclear ownership of platforms, fragmented data governance, and inconsistent data standards [5]. While various organizations and institutions aim to generate comprehensive global overviews of respiratory pathogen circulation and burden, the involvement of numerous stakeholders has led to a fragmented landscape with overlapping efforts, and limited interoperability and collaboration [6, 7].

To address these challenges, the Center of Excellence for Respiratory Pathogens (CERP), in collaboration with Netherlands Institute for Health Services Research (Nivel), organized a two-day workshop in Lyon, France, in 2025. The workshop brought together dashboard creators, developers, and users to discuss the current landscape of respiratory pathogen surveillance. The first day focused on the creators and developers of the dashboards, while the second centered on the perspectives, needs and expectations of their users.

Two main outcomes emerged. First, thanks to the wide range of participants — representing local, regional, national, global, and private initiatives (Appendix 1), — the workshop provided a unique multi-stakeholder overview of existing respiratory pathogen dashboards [8–16]. This allowed for comparison of their goals, structures, and challenges, as summarized in Table 1. Second, the expert discussions throughout the workshop fostered mutual learning and exchange, helping to identify shared challenges and explore future directions for improvement. This report aims to make these insights accessible to data producers, dashboard developers and users of respiratory pathogen surveillance tools. While many of the lessons discussed are relevant across different types of dashboards, they are particularly applicable to tools that aggregate and publish heterogeneous data from multiple sources to support the epidemiological monitoring of large-scale diseases, such as those caused by respiratory pathogens.

**Table 1:** Summary of the presented dashboards (and bulletins) for respiratory pathogen surveillance

	**Organization**	**Purpose**	**Intended audience**	**Viruses**	**Data sources**	**Format/Interface**
**Local**						
Bulletin Épidémiologique Hospices Civils de Lyon (BEHcl)	Hospices Civils de Lyon (HCL)	Weekly epidemiological bulletin summarizing local hospital and laboratory data	Local healthcare professionals; 660 recipients per request	Influenza RSV SARS-CoV-2 Other respiratory viruses	^a^PCR data from HCL’s hospital laboratory information system	Three-page PDF bulletin combining manual texts and selected graphs emphasizing clarity, brevity, and visual impact
**National/Regional**						
Santé Publique France (SPF) ARI surveillance system	SPF: national public health institute of France	National ARI surveillance, consolidated and reported weekly	National and regional policymakers Epidemiologists Clinicians General public	Influenza Rhinoviruses RSV SARS-CoV-2	Mortality data (electronic death certificates) Syndromic hospital data (ICU sentinel network) Emergency visits Virological data (National Reference Laboratory) Wastewater surveillance (SUM'Eau)	PDF-bulletin report Current and future development of automated data triangulation and user-friendly interfaces
Ontario respiratory virus tool (ORVT)	Public Health Ontario (PHO)	Weekly-updated, centralized influenza surveillance via a comprehensive, interactive interface	Public facing: PH authorities Policymakers Researchers HCPs	Influenza RSV SARS-CoV-2	Outbreak data (Public Health Case and Contact Management Solution (CCM)), Integrated Public Health Information Systems (iPHIS) Hospital capacity (Ontario MoH Daily Bed Consensus) Laboratory surveillance systems Population data (Statistics Canada)	Interactive dashboard with user customization: explore trends overtime, strain data, filtration by geography and other variables Also translated to French
ERVISS	ECDC and WHO jointly developed surveillance system for respiratory pathogens across EU/EEA WHO Regional Office for Europe	Weekly updated to: Monitor spread, intensity, and timing of virus activity Track severity, risk factors, and impact on health systems Identify and monitor changes in circulating strains, including emerging viruses	Public facing: PH authorities Policymakers Researchers HCPs	Influenza RSV SARS-CoV-2	Standardized reporting by country contact points to TESSy: Syndromic surveillance in primary and secondary care Multiplex virological testing Sentinel laboratory networks Genomic surveillance	ERVISS provides interactive, user-friendly presentation: Plots and tables Free text epidemiological summaries Customization options
**Global**						
Global Influenza Surveillance and Response System (GISRS): FluNET FLUID	World Health Organization (WHO)	Global, standardized tools for virologic (FluNET) and syndromic (FluID) surveillance	Dashboards range from public-facing to internal, and country-level support	Influenza RSV SARS-CoV-2	FluNET: National Influenza Centers (NICs) and national reference laboratories report weekly laboratory-confirmed virological data FluID: National health authorities compile and report weekly clinical and syndromic surveillance data on SARI and ILI	FluNET: Interactive dashboard FluID: Reporting platform
Global Influenza Hospital Surveillance Network (GIHSN)	Foundation for Influenza Epidemiology	Improve comparability of clinical and virological surveillance data across countries and regions for hospitalized ILI Inform global PH decision-making and vaccine strain collection Link virus characteristics to disease severity	Public-facing	Mainly Influenza RSV SARS-CoV-2, but increasingly all respiratory viruses available through multiplex PCR	Institutions apply to catalytic style grants About 120 hospitals across 25 sites worldwide. 30,000 + tested patients yearly Year-long, prospective surveillance studies, systematic testing standardized protocol Annual reporting of results. Repost for WHO vaccine composition meeting	Visual, interactive dashboard (moving toward real time) Based on standardized clinical datasets Emphasize linking epidemiological, clinical, and genome sequencing data
**Global (Research/Institutional)**						
EPI Live!	CERP	Monitor live (weekly-update) global epidemiological trends, in a historic perspective Facilitate access to the data, compared to access to the multiple existing dashboards and by providing contextual information to facilitate data interpretation	Public facing: Scientists HCPs PH professionals People working in epidemiological monitoring	Influenza RSV	Web-scraping of publicly available global, national, and regional sources (WHO, CDC, and national institutes) Laboratory-confirmed cases and positivity rate, plus specific indicators depending on the data source (regarding disease activity, strain circulation, severity, or mortality)	Interactive website (multiple independent pages, ability to register) providing a worldwide, country, and sub-country (depending on country) views. A user can compare geographies and periods and register for some additional features including bookmarking capabilities and automated weekly mails based on epidemic evolution. Developed to provide contextual information (at graph, country, and data source levels) to support a broader audience usage
FluCov	Netherlands Institute for Health Services Research (Nivel)	Monitoring co-circulation of SARS-CoV-2 and Influenza	Public-facing	Influenza SARS-CoV-2	Combining WHO FluNet (influenza) and Our World in Data (SARS-CoV-2) for positive cases Covering 25 countries based on geographic diversity	Interactive dashboard with filtration based on time period, virus, and geographical region Monthly bulletin reports, bi-weekly activity reports, and seven scientific publications
ReSViNet	ReSViNet Foundation	Provide simplified, standardized, and centralized, monthly global trends in RSV circulation Raise awareness Monitor RSV seasonality Compile up-to-date registry of RSV surveillance systems	Public-facing: Policymakers PH professionals Researchers Pharmaceutical stakeholders	RSV	Partially-automated scraping combines data from ERVISS, WHO, FluNet, and national datasets Weekly positivity rates calculated per country (as extracted), based on available country data and averaged across two full seasons (seasonal benchmarks) and standard-deviation	Interactive dashboard with filtration based on time period, geographical region, and source
AIOLOS	AIOLOS consortium Franco-German initiative funded by FR and GER governments	Real-time, early-warning and response tool for respiratory pathogen outbreaks Support scenario-planning and decision-making	Mostly PH and HPCs	Respiratory virus	Syndromic data (based on ambulatory care software or hospitals) Virological data Non-traditional data sources (mobility data, social media data, wastewater surveillance)	Interactive dashboard including AI-powered models and insights based on surveillance data
**Global (Private)**						
OneID	AirFinity	Real-time infectious disease intelligence and simulation tool	Restricted access: Range of users, collaborators, and customers	Over 160 human and zoonotic diseases	Over 7,000 data sources (85% public): (Social) media NGOs Scientific publishers Governmental organizations, etc	Three types of dashboards support varying levels of decision-making Insight-driven

*Abbreviations*: *ARI* acute respiratory infection, *BEHcl Bulletin Épidémiologique des Hospices Civils de Lyon*, *CDC* Centers for Disease Control and Prevention, *CERP* Centre d’Épidémiologie et de Recherche en Population, *CCM* Public Health Case and Contact Management Solution, *ECDC* European Centre for Disease Prevention and Control, *EEA* European Economic Area, *ERVISS* European Respiratory Virus Surveillance Summary, *FR* France, *GER* Germany, *GIHSN* Global Influenza Hospital Surveillance Network, *HCL* Hospices Civils de Lyon, *HCPs* healthcare professionals, *ICU* intensive care unit, *iPHIS* Integrated Public Health Information System, *ILI* influenza-like illness, *MoH* Ministry of Health, *NICs* National Influenza Centers, *Nivel* Netherlands Institute for Health Services Research, *ORVT* Ontario Respiratory Virus Tool, *PCR* polymerase chain reaction, *PH* public health, *PHO* Public Health Ontario, *RSV* respiratory syncytial virus, *SARI* severe acute respiratory infection, *SPF Santé publique France*, *SUM’Eau* French wastewater surveillance program (*Surveillance Unifiée des Maladies par l’Eau*), *TESSy* The European Surveillance System, *WHO* World Health Organization

**Fig. 3:**
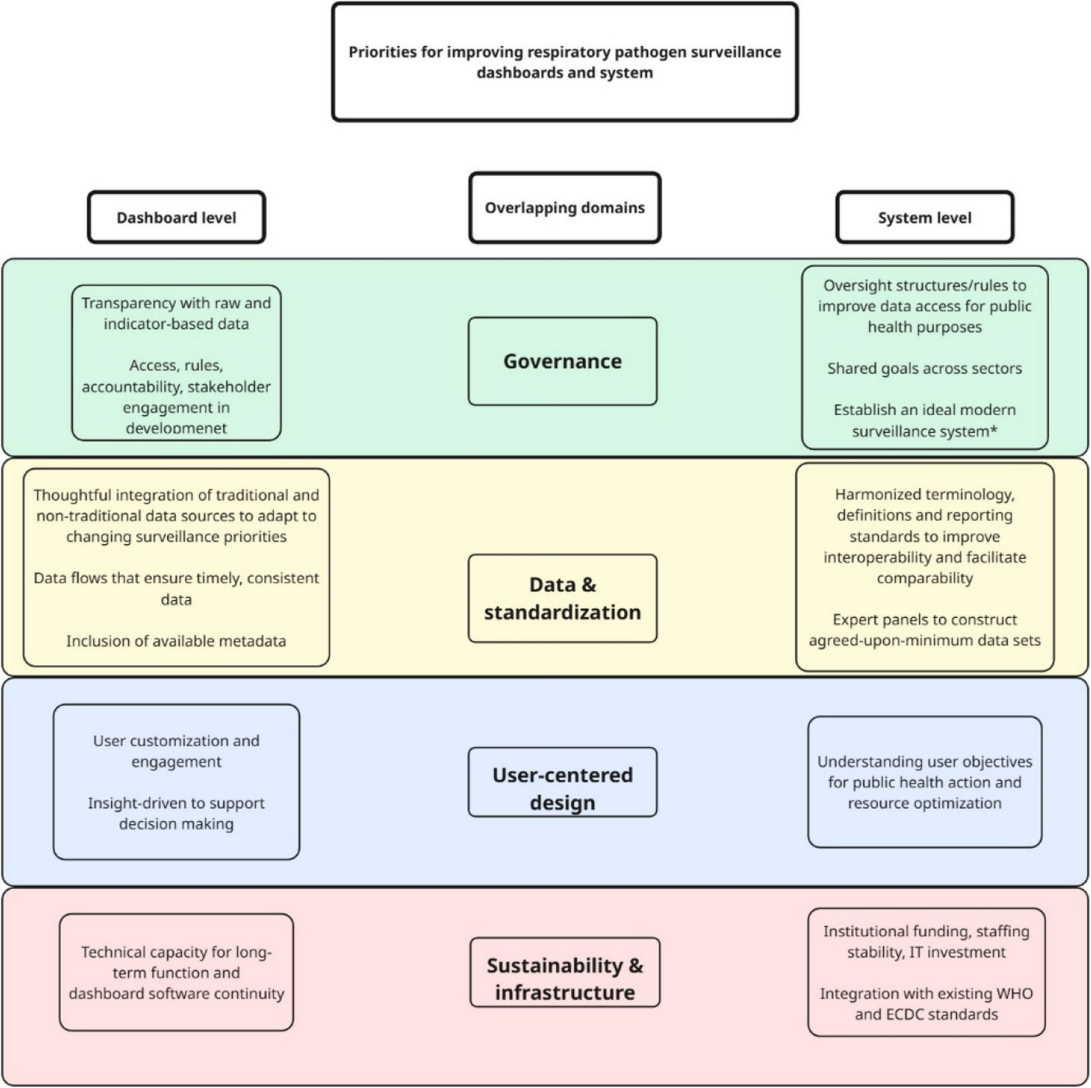
Respiratory pathogen surveillance and priorities within the domains of dashboard development at the system level. Overlapping domains of priorities for respiratory pathogen surveillance relate to ensure that dashboards are technically functional, user-centered systems installed within resilient, interoperable surveillance systems. *Such as: i) incidence rates based on primary care sentinel surveillance with defined catchment populations; ii) standardized symptom definitions; iii) hospital-based severe acute respiratory infections (SARI) surveillance; iv) geographic and demographic representativeness; v) comprehensive virological testing of specimens; vi) rich metadata, and; vii) inclusion of additional data sources (e.g. vaccine coverage, seroprevalence, wastewater surveillance)

The diverse array of expert participants from various public and private organizations gave way to fruitful discussions spanning topics across domains of user-centeredness, data and standardization, sustainability and infrastructure, and governance. Of high importance is the need for improved data quality, transparency, interoperability, and collaboration. Inherently, improving respiratory pathogen surveillance dashboards is highly interlinked with improving the broader field of surveillance. This report acknowledges that overlap, with the aim of keeping the focus on dashboards themselves. Figure 3 highlights major actionable recommendations for strengthening the field of respiratory pathogen surveillance and dashboards.

## Building dashboards—what is needed, challenges, and opportunities

### Objectives, data sources, and target audiences

The data displayed in dashboards should align with the platform’s overall aims—whether for traditional forms of surveillance, expanding data access, facilitating research, healthcare improvement, early warning, or pandemic preparedness. Sentinel and non-sentinel reporting and data collection are a major component of traditional surveillance, are generally reliable (by design), but can be slow, as they often rely on clinical, laboratory-confirmed detections. In contrast, early warning and signal detection systems often additionally include non-traditional data sources, such as wastewater surveillance which can precede hospital trends by 1–2 weeks [17], along with qualitative signals from media and news sources [18]. While non-traditional data streams can provide more rapid insights, they may be less specific or reliable [19]. Workshop participants discussed the need to incorporate various forms of data, depending on the objective of the dashboard, as well as the target audience. Participants agreed that such sources of quick information are supplementary, and dashboards must clearly communicate data limitations and context. Faster, less validated data sources contribute to overall data interpretation, but must be interpreted with caution.

The intended audience also shapes dashboard design, as there is no one dashboard that can fit all audiences with differing expectations. Moreover, efficiency, accessibility, and sustainability of interactive systems benefits largely from human-centered design approaches [20]. This spectrum was revealed in the workshop. For example, BEHcl reports weekly summaries for quick, data-driven decision-making in health care settings, ReSViNET aims to make simplified visualizations for the general public, and EPI Live! increases understanding of indicators to audiences with broad epidemiological expertise. Certain contextual elements, or restricted access and customization may vary to minimize the risk of interpretation. For example, epidemiologists and medical professionals are less prone to misinterpret data, whereas decision-makers without a technical background likely benefit from additional contextual notes or expert guidance to ensure accurate and meaningful interpretation of findings. Due to this uncertainty, some dashboards choose to disable country-to-country comparisons due to the concern that users might overlook important context differences, e.g. differences in surveillance systems. Inclusion of context variables into dashboards is feasible, but time-consuming. Developers must balance transparency and openness with the risk of misinterpretation.

### Technical components and user friendliness

When building dashboards, several standard components are common. Most platforms allow users to interact with map and chart visualizations by filtering data based on geography, time period, and data source. A major challenge highlighted by public dashboard presenters was staff shortage: maintaining dashboards in real-time is not fully automated and often relies on very small dedicated teams—sometimes just a single individual. This exposes the vulnerability of surveillance in general and constitutes the “Achilles heel” in public surveillance systems that depend on few individuals supported by insufficient funds. Many developers also expressed interest in expanding their bulletins or dashboards to: i) integrate data from multiple pathogens and non-sentinel sources; ii) broaden viral coverage; iii) enhance country-level summaries, and; iv) improve user customization. While technically feasible, such expansions raise new issues such as: maintaining clarity and usability; interoperability barriers; defining meaningful thresholds and trends, and; compiling and interpreting data from heterogeneous systems. Private, custom-built dashboards face different trade-offs. They offer strong control over data flows, tailoring to users, and flexible infrastructure. However, challenges surrounding access to public data and limited direct business interest comparatively to the investment remain.

On a more technical level, IT infrastructure is often overlooked. Developers stressed the need to include IT from the beginning to ensure maintainability and sustainability of these complex systems. The discussion on automation, such as by using application program interfaces (APIs) for automated data uploading, focused on improving timeliness and efficiency, while balancing the need for appropriate oversight. Automation does not necessarily imply the use of artificial intelligence (AI), nor does it mean processes must run without supervision. Approaches such as human-in-the-loop systems were seen as essential to managing risks and maintaining quality. Looking ahead, participants saw potential for selectively integrating AI to enhance user interaction. For example, through intelligent front-end prompting that allows users to query indicators directly. In general, strengthening IT capacity, automation, and AI may help alleviate staff shortages, reduce running costs, and enhance the responsiveness, flexibility and sustainability of respiratory dashboards.

### Dashboard quality in relation to data accessibility

Key determinants of dashboard quality discussed in the workshop were the availability, timeliness, and completeness of the underlying data, and sources processed and transformed within the dashboard. Many current dashboards rely on a limited set of public data sources (e.g. fluNet/FluID, ERVISS, and national datasets), each with inherent limitations that cascade into the visualized indicators. Such limitations include gaps in geographic coverage, differences in reporting standards, reduced data availability for certain countries, and inconsistencies in timeliness of reporting and/or surveillance approaches. One way to improve the quality of dashboards is through promoting data accessibility, including in areas where data are scarce. On one hand, dashboard producers are increasingly expected to be transparent not only with indicator-based or aggregated data, but also with their raw, analyzable data. On the other hand, obtaining access to existing data remains a major obstacle for dashboard producers. Although much of the information exists and is being stored, stakeholders often encounter significant barriers and opaque access procedures, extending also to metadata. Legal and regulatory frameworks, mainly General Data Protection Regulation (GDPR) in Europe, alongside national privacy and data protection laws, pose major restrictions for countries and institutions [20]. Additionally, data licensing, compliance monitoring, and institutional gatekeeping are recurring challenges. Data holders may create the appearance of openness, encouraging users to request access, only to later fail to respond to inquiries or impose restrictive conditions such as ethical approval processes. Looking forward, presenters stressed the need for stakeholders to reduce barriers by: working on data sharing agreements, promoting the use of summary data and statistics, and improving transparency in data flows. Moreover, respiratory pathogen surveillance dashboards would benefit from GDPR-compliant, open access systems promoting responsible data usage for public health purposes [21]. Participants also debated the tension between privacy protection and public‑health utility. Privacy legislation is indispensable, yet it can hinder timely sharing when rapid situational awareness is critical. Achieving an appropriate balance will require stronger data‑governance frameworks that combine ethical safeguards with mechanisms guaranteeing access to key datasets for public‑health purposes, irrespective of the requester’s affiliation.

Power dynamics surrounding data sharing and management heavily restrict the ability to use existing data. Some public actors and small companies often lack the resources to collect or access high-quality data, while other organizations may benefit from exclusive data-sharing agreements with certain partners or countries. Additionally, the private sector encounters similar barriers; industry representatives described restrictions on raw data use for commercial re-use. Health record holders and data intermediaries operating between the public and private sectors can further exacerbate inequality by acting as gatekeepers and setting high prices for key datasets. Finally, participants underscored that technical literacy and contextual understanding of data sources are as important as access itself: knowing where data is coming from, how data are generated, transformed, and curated is fundamental to building meaningful and trustworthy surveillance dashboards.

### Dashboard quality in relation to standardization

Another impacting factor on dashboard quality emphasized in the discussions was that of standardization, whether regarding data collection or uniformity in data entry and reporting. Regional and global organizations such as the WHO and ECDC play a central role in setting reporting and management standards. RSV surveillance, for example, still varies widely across countries, often conducted through nationally initiated systems that remain works in progress. In this context, the 2024 publication of the Integrated Surveillance Standards [22] was highlighted, which defines the minimum data requirements for sentinel sites, patient sampling, and laboratory reporting. In particular, WHO is encouraging countries to share more disaggregated, case-based data, which are crucial for estimating disease burden, linking clinical and genomic data, and generating real-world evidence on vaccine effectiveness and impact.

The importance of standardization on dashboard quality extends to finer technical and semantic levels. Incorrect and inconsistent categorization—for instance, conflating gender with biological sex, or race with ethnicity—reveals a lack of standardization of terminology in various databases. Additionally, arbitrary implementation of ‘small number suppression’ policies (often related to privacy) may obscure important information when thresholds are chosen without sufficient explanation. Vague categories such as ‘missing’ or ‘unknown’ categories further complicate interpretation.

Participants agreed that transparent, well‑documented metadata standards and consistent application of terminology are indispensable for building trust in dashboards. Without clarity on how data are defined, aggregated, and suppressed, users risk misinterpretation and reduced comparability across countries and systems.

## Dashboards for public health action

### Data integration

Cycles of producing, consolidating, visualizing, and contextualizing data are inherently complex, yet essential for enabling timely, data-based and effective public health decision-making. Robust data integration is needed to inform resource or intervention allocation, evaluate interventions, guide communication strategies, and assess real-time risks [23]. When multiple data sources are triangulated—combining clinical, laboratory, environmental, and behavioral signals—they can create higher-quality indicators. Some experts underscored the value of voluntary redundancy of data points, where diverse overlapping sources confirm the same trends. Repeated trend confirmation increases confidence in the findings and strengthens model accuracy, leading to better informed public health decisions for various audiences. For example, from the perspective of health system decision-makers, policy-actionable evidence integrates data from multiple domains – including but not limited to hospital and ICU occupancy rates, health workforce capacity, and supplies of vaccines or therapeutics. Technological advances in forecasting models can address the complexity of both data landscape and decision-making, providing clarity on specific questions such as: ‘What short-term benefit can we expect from deploying a specific vaccine?’, or ‘How many vaccines should be stockpiled in my country for the next season?’.

Importantly, dashboards should be seen as living systems that evolve constantly to reflect changing objectives and emerging data sources. For example, environmental and animal surveillance are becoming increasingly relevant, especially for tracking IVPPs, and understanding transmission zones [24]. This increasing intersectionality further underscores the need for stronger coordination across sectors, both nationally and internationally, as well as better harmonization between human and animal health domains. There was also a general consensus among participants on the need to push towards more integrated dashboards and platforms, capable of addressing the complexity of multi-pathogen surveillance in almost real time. Moreover, modularity and adaptability in dashboards allows for more feasible, short-term improvements for respiratory pathogen surveillance, whereas improving the entire surveillance system is a more complicated task.

### Collaboration

While respiratory pathogen surveillance inherently spans multiple sectors and disciplines, workshop participants expressed concern over the consequences of lack of collaboration. Persistent disciplinary silos were identified as significant barriers and exist between: human, animal, and environmental health sectors; public health specialists, clinicians, and virologists; and between public and private actors. Yet, many goals are aligned across stakeholders. For example, recommendation and reimbursement for vaccines is directly related to their demonstrated health value, thus public and private sectors share an interest in measuring disease burden, supporting increased vaccine uptake, and maximizing population-level protection. Effective surveillance dashboards (and systems) are essential not only for seasonal preparedness and outbreak detection, but also to guide public health measures and support vaccine development, including the refinement of antigen design based on circulating strains. Participants noted that public health value is often a key driver of commercial value, and that building a more cooperative mindset could unlock surprising shared benefits.

Finding the “sweet spot” between potential conflicts of interest and data governance could help mediate tensions around data sharing and access, improve transparency, and ultimately enhance surveillance capacity. Suggestions to improve collaboration included: involving key private-sector data providers to help unlock access to underutilized datasets; creating multi-stakeholder forums to support real-time, evidence-based decision-making; and promoting participatory dashboard development, where relevant actors co-create or contribute to shared surveillance platforms. A more cooperative approach—rooted in transparency, shared objectives, and mutual benefit—has the potential to reduce fragmentation and improve the global respiratory pathogen surveillance ecosystem.

## Conclusion: cross-cutting themes and open questions

Throughout the workshop, several themes emerged that cut across technical, governance, and operational dimensions of respiratory pathogen dashboards. First, participants consistently emphasized the tension between speed and accuracy. While near-real-time data can enable timely response, it often comes at the expense of completeness, validation, and interpretability. Achieving the right balance (according to the dashboard’s ultimate objective), and communicating data limitations clearly, remains a shared challenge across platforms. Second, there was widespread agreement on the importance of governance, funding models, and collaboration. Dashboards often rely on small teams and fragile infrastructures, raising concerns about sustainability. Some participants called for stronger coordination mechanisms, better integration between public and private actors, and transparent data-sharing frameworks that serve public health goals while protecting individual rights and commercial sensitivities. This is a delicate balance, as the population may lose trust in the public health system, for example in vaccine promotion, if the collaboration with private actors is perceived as a conflict of interest. On the other hand, technologies and analytics (e.g. AI) have revolutionized the landscape, and the public sector cannot always cope alone given the level of agility and resources it requires. Ignoring this fact incentivizes the development of parallel systems which can also potentially affect public trust. Third, the need for standardization was discussed not only in relation to technical data fields, but also terminology, data presentation, and thresholds for alerts or trends. Without common standards, dashboards risk being misinterpreted or losing interoperability.

Several open questions were raised but remain unresolved: i) What is the optimal governance model to ensure long-term sustainability and trust in global respiratory pathogen dashboards? ii) How can underutilized data, especially from the private sector or proprietary systems, be made more accessible for public health benefit? iii) Can integrated, multi-pathogen platforms be achieved without overwhelming users or compromising specificity? iv) What role could regional or global consortia play in harmonizing efforts and funding innovation?

These cross-cutting issues call for continued dialogue and coordinated action. As respiratory surveillance becomes more digitized, the field must ensure that technical progress is matched by ethical, strategic, and user-centered innovation.

The workshop highlighted the critical need to reduce fragmentation in respiratory pathogen surveillance by strengthening interdisciplinary collaboration and data integration. Participants emphasized that with collaboration, we can improve data governance, enhance standards and access, and promote transparency and accountability in public health. Importantly, data ownership and governance structures must support equitable access, ensuring fair data ownership while respecting legal and ethical frameworks. Standardization efforts should be both top-down (lead by organizations like WHO and ECDC) and bottom-up, with national public health institutes, academic networks, and developers contributing to shared data and design standards. Large, public–private collaborative initiatives such as the Innovative Health Initiative (IHI) may provide promising models for unifying and advancing these goals, provided they are able to engage EU or Member States public agencies together with private sector players. Complementary regional and national consortia should also be considered to complement large-scale efforts in sustaining system-level improvements over time, which may require further funding and resources. A synthesis of actionable recommendations discussed during the workshop is presented in Fig. 3.

## Data Availability

Not applicable. No primary data were generated for this report. The manuscript summarizes discussions and insights from a workshop; all relevant information is included within the article and its supplementary materials.

## Appendix 1

### Participant institutions and workshop program

**Table:**

**Participating organizations**
1. Center of Excellence in Respiratory Pathogens (CERP) 2. Netherlands Institute for Health Services Research (Nivel) 3. Hospices Civils de Lyon (HCL) 4. TeamWork 5. University of Florence 6. Santé Publique France (SPF) 7. European Center for Disease Control (ECDC) 8. World Health Organization (WHO) 9. Artificial Intelligence TooLs for Outbreak Detection and ResponSe (AIOLOS) 10. AirFinity 11. Direction Générale de la Santé (DGS) 12. ReSViNET Foundation 13. Sanofi
**Presentations Day 1**
1. EpiLive Dashboard/Teamwork 2. FluCov Dashboard 3. ReSViNET Dashboard 4. BeHCL (HCL bulletin) 5. AIOLOS project outcomes and next steps 6. AirFinity OneID 7. ECDC: The EU surveillance system (TESSy) & European Respiratory Virus Surveillance Summary (ERVISS) 8. Ontario Respiratory Virus Tool (ORVT) 9. Global Influenza Hospital Surveillance Network (GHISN) Dashboard
**Presentations Day 2**
1. Nivel project on Influenza Viruses with human Pandemic Potential (IVPPs) 2. Interdisciplinarity in the field of respiratory pathogen surveillance 3. From data to outputs to insights, and back again: a journey toward dashboard building 4. What standards do public health authorities set for surveillance of acute respiratory infections 5. What does the Ministry of Health (MoH) seek in dashboards? When is a dashboard a good dashboard 6. What information does the WHO look for in dashboards on flu, RSV, and COVID? 7. What does the industry seek for in dashboards on flu, RSV, and COVID-19? 8. Sick of surprises? Smarter insights for safer populations

## Abbreviations

AI: Artificial intelligence
API: Application program interface
BEHcl: Bulletin Épidémiologique des Hospices Civils de Lyon
CDC: Centers for Disease Control and Prevention
CERP: Centre d’Épidémiologie et de Recherche en Population
ECDC: European Centre for Disease Prevention and Control
EEA: European Economic Area
ERVISS: European Respiratory Virus Surveillance Summary
FR: France
GDPR: General Data Protection Regulation
GER: Germany
GIHSN: Global Influenza Hospital Surveillance Network
HCL: Hospices Civils de Lyon
HCPs: Healthcare professionals
ICU: Intensive care unit
IHI: Innovative Health Initiative
iPHIS: Integrated Public Health Information System
ILI: Influenza-like illness
IVPPs: Influenza viruses with human pandemic potential
MoH: Ministry of Health
NICs: National Influenza Centers
Nivel: Netherlands Institute for Health Services Research
ORVT: Ontario Respiratory Virus Tool
PCR: Polymerase chain reaction
PH: Public health
PHO: Public Health Ontario
RSV: Respiratory syncytial virus
SARI: Severe acute respiratory infection
SPF: Santé publique France
SUM’Eau: Surveillance Unifiée des Maladies par l’Eau
TESSy: The European Surveillance System
WHO: World Health Organization
ARI: Acute respiratory infection
CCM: Public Health Case and Contact Management Solution
