# Correction: Respiratory virus surveillance in the post-pandemic era: challenges and opportunities for dashboard-based public health action

**DOI:** 10.1186/s12919-026-00372-6

**Published:** 2026-04-02

**Authors:** Adrienne Halley, Caroline Schneeberger, Foekje F. Stelma, Aby Ba Diallo, Ombeline Jollivet, Bronke Boudewijns, Marie-Noëlle Billard, Julika Frome, Jean-Sebastien Casalegno, Katharina B. Lauer, Cédric Mahé, Erica Dueger, Marco Del Riccio, Alexandre Descamps, Anna Maisa, Siddhivinayak Hirve, Saverio Caini, Marta C. Nunes

**Affiliations:** 1 Netherlands Institute for Health Services Research (Nivel), Utrecht, the Netherlands; 2 Amsterdam University Medical Center (AUMC), Amsterdam, the Netherlands; 3 Center of Excellence in Respiratory Pathogens (CERP), Hospices Civils de Lyon (HCL) and Centre International de Recherche en Infectiologie (CIRI), Équipe Santé Publique, Épidémiologie Et Écologie Évolutive Des Maladies Infectieuses (PHE3ID), Inserm, ENS de Lyon, Université Claude Bernard Lyon, Lyon, France; 4 Sanofi Vaccine, Lyon, France; 5 Department of Pediatric Infectious Diseases and Immunology, Wilhelmina Children’s Hospital, University Medical Center Utrecht, Utrecht, Netherlands; 6 ReSViNET Foundation, Zeist, The Netherlands; 7 Laboratoire de Virologie, Centre National de Référence Des Virus Des Infections Respiratoires, Institut Des Agents Infectieux, Hospices Civils de Lyon, Lyon, France; 8 Airfinity Ltd, London, UK; 9 University of Duisburg-Essen, Essen, Germany; 10 Department of Health Sciences, University of Florence, Florence, Italy; 11 Directorate General for Health, Ministry of Health, Paris, France; 12 Department of Infectious Diseases, Santé Publique France, Saint-Maurice, France; 13 Global Influenza Programme, World Health Organization, Geneva, Switzerland

**Correction: BMC Proc 20 (Suppl 13), 10 (2026)**

**https://doi.org/10.1186/s12919-026-00368-2**

Following publication of the original article [1], the authors identified an error in the author name of Alexandre Descamps.

The incorrect author name is: Alexandre Descamp

The correct author name is: Alexandre Descamps

Affiliation 11 has been corrected to: Directorate General for Health, Ministry of Health, Paris, France.

Further to this, the authors noted some grammatical errors in table 5. The last column of “EPI Live!” in the section “Global (Research/Institutional)” should read:

Interactive website (multiple independent pages, ability to register) providing a worldwide, country, and sub-country (depending on country) views. A user can compare geographies and periods and register for some additional features including bookmarking capabilities and automated weekly mails based on epidemic evolution. Developed to provide contextual information (at graph, country, and data source levels) to support a broader audience usage.

In the third paragraph of the Introduction, there is an erroneous bracket which needs to be removed. The first sentence of this paragraph should read: Two main outcomes emerged. First, thanks to the wide range of participants — representing local, regional, national, global, and private initiatives (Appendix 1) …

The author group above has been updated and the original article [1] has been corrected.
